# Sigmoid (S-shaped) cross-fused renal ectopia associated with calculus in a 3-year-old child: A case report and literature review

**DOI:** 10.1016/j.eucr.2026.103468

**Published:** 2026-05-12

**Authors:** Turyalai Hakimi, Edris Nejrabi, Zamaryalai Hakimi, Mansoor Aslamzai, Mohammad Anwar Jawed

**Affiliations:** a Department of Pediatric Surgery, Kabul University of Medical Science, Maiwand Teaching Hospital, Kabul, Afghanistan; b Department of Nutrition, Kabul University of Medical Science, Kabul, Afghanistan; c Department of Neonatology, Kabul University of Medical Science, Maiwand Teaching Hospital, Kabul, Afghanistan

**Keywords:** Renal ectopia, Sigmoid, Stone, Hydronephrosis, Nephrolithotomy

## Abstract

Sigmoid (S-shaped) crossed-fused renal ectopia is a rare congenital anomaly, infrequently complicated by urolithiasis. We report a 3-year-old male child presenting with abdominal pain, diarrhea, and intermittent fever, previously diagnosed with a renal stone. Imaging revealed a 13-mm pelvic stone in the lower ectopic kidney with moderate hydronephrosis; laboratory tests were normal. The patient underwent open nephrolithotomy via a right modified Gibson incision, achieving complete stone removal through a small avascular parenchymal incision. Recovery was uneventful, and stone composition analysis showed mixed calcium oxalate and calcium phosphate stone. Careful imaging and individualized surgical planning are essential in complex renal anatomy.

## Highlights

• **Sigmoid crossed-fused renal ectopia is a rare anomaly, occasionally presenting with urolithiasis in children.**
• **Preoperative imaging is essential to localize stones and plan safe surgical access.**
• **Endourological approaches are preferred, but open nephrolithotomy via modified Gibson incision allows safe stone removal in anatomically complex kidneys.**
• **Postoperative recovery can be excellent with preservation of renal parenchyma in pediatric patients.**
• **In** **developing countries, stone analysis and nutritional surveillance help prevent recurrence.**

## 1 Introduction

Crossed-fused renal ectopia (CFRE) is an uncommon congenital renal malformation characterized by the presence of both kidneys on the same side of the body. The estimated incidence is approximately 1 in 7000 individuals.^1^ The majority of cases remain clinically silent and are detected incidentally during imaging performed for unrelated reasons. However, this anatomical anomaly is associated with an increased risk of urinary tract obstruction, recurrent urinary tract infections (UTIs), nephrolithiasis, and ureteropelvic junction obstruction (UPJO), predominantly arising from mechanical factors related to the anomalous renal anatomy.^2^^,^^3^

## 2 Case presentation

A 3-year-old male child was brought to our pediatric surgery unit with a previously diagnosed renal stone in the ectopic component of a right-sided sigmoid CFRE. According to the patient's grandfather's explanation the patient had been suffering from nonspecific abdominal pain, diarrhea, and intermittent fever. The patient was born through vaginal delivery at home traditional setting from a couple with positive consanguinity.

The patient sought several medical centers for above-mentioned symptoms with varying degrees of improvement. In the public hospital, the attendant physician ordered intravenous urography (IVU) which confirmed stone in the ectopic component of a right-sided sigmoid CFRE. After the diagnosis, the patient was discharged with conservative recommendations, but showed no improvement.

**Fig. 1:**
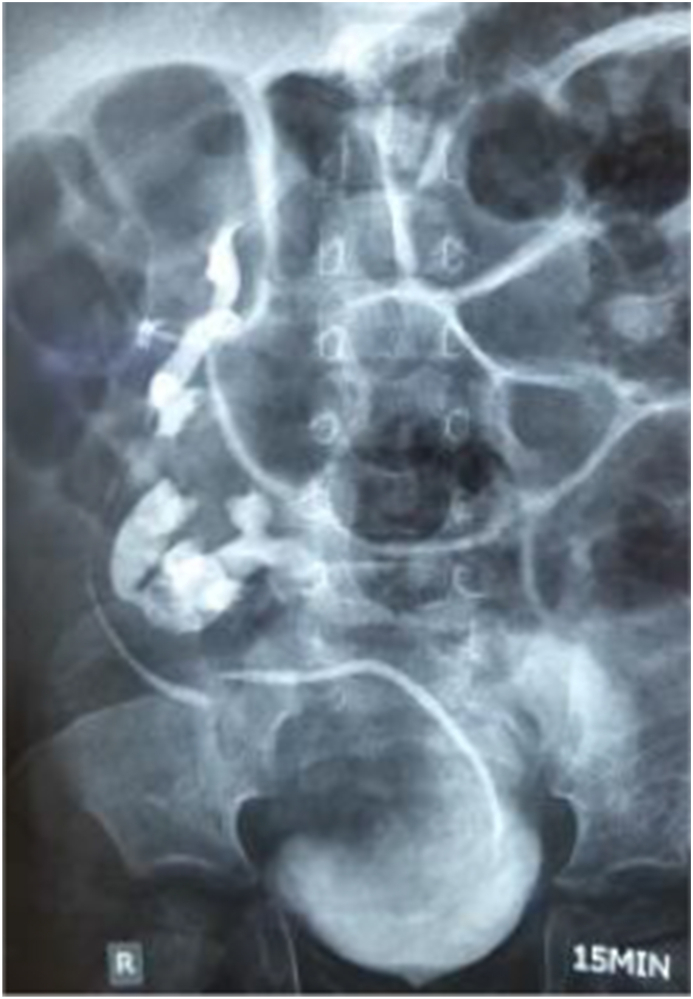
Intravenous urography (IVU) demonstrating S-shaped crossed fused renal ectopia with a calculus in the ectopic left kidney.

On admission to our surgery unit, the patient had already had IVU confirming the diagnosis of renal stone in the left ectopic kidney (Fig. 1). To achieve the precise size of the stone, our team ordered ultrasonography (USG), which revealed a 13mm stone in the pelvis of the ectopic (left) lower kidney causing moderate hydronephrosis. All routine and biochemical laboratory examinations were normal reported by our laboratory department.

**Fig. 2:**
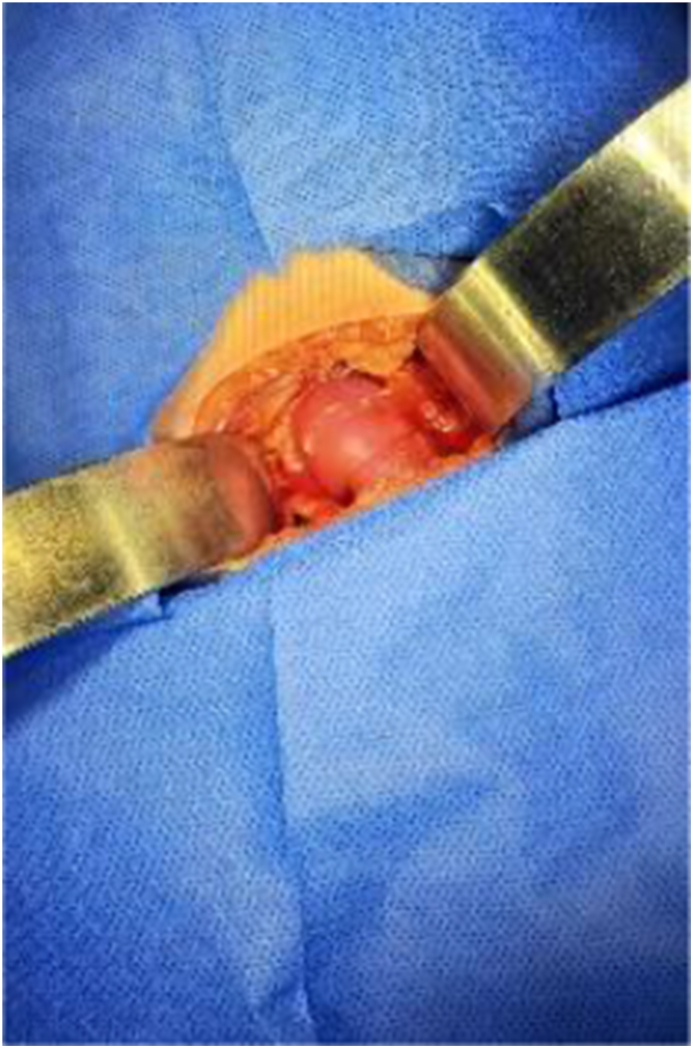
Intraoperative view depicting S-shaped crossed fused renal ectopia.

**Fig. 3:**
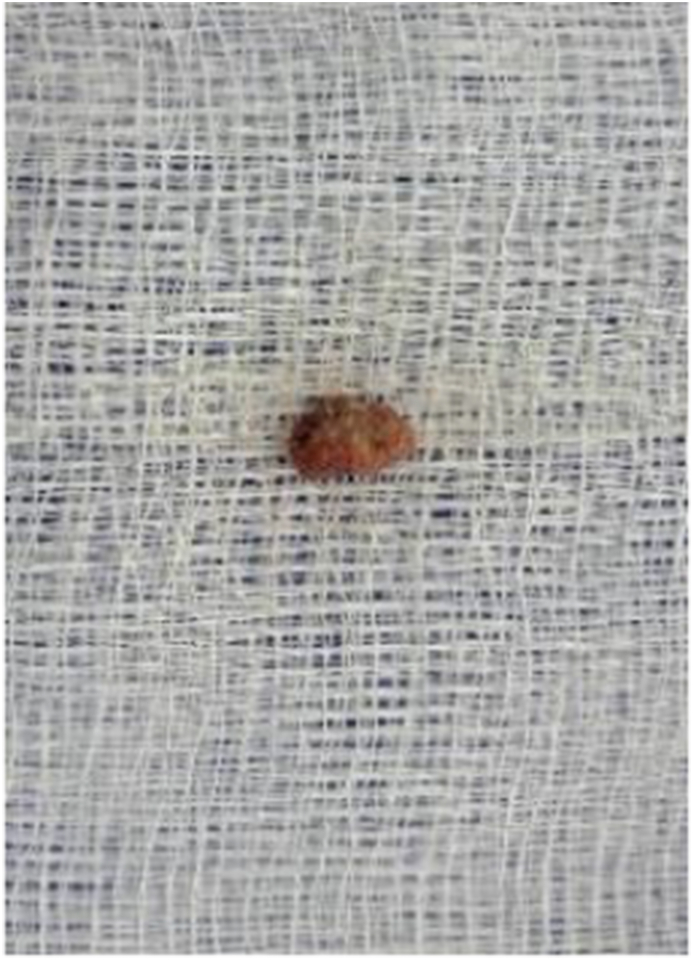
Removed calculus.

The patient was scheduled for definite treatment. Our team approached the stone through a right modified Gibson incision to achieve extensive exposure and access to the ectopic kidney (Fig. 2). The kidney was accessed and was found to have a small stone in lower pole of the ectopic kidney. Initially, pyelolithotomy was planned to preserve the renal parenchyma. However, because the stone was located very close to the thin cortex of the ectopic kidney, it was removed via a nephrolithotomy through a small incision in an avascular area of the kidney (Fig. 3). A perirenal catheter was placed and the wound was closed in layers. The stone analysis reported (calcium oxalate dihydrate 75%, calcium oxalate monohydrate 25%, and calcium phosphate 5%). The three days hospital stay was smooth and the patient was discharged in satisfactory condition with supportive and nutritional surveillance.

## 3 Discussion

CFRE is an uncommon congenital renal malformation characterized by both kidneys being positioned on the same side of the body, while the ureter of the ectopic kidney retains its normal insertion into the urinary bladder. This anomaly is classified into two distinct forms: crossed unfused renal ectopia and CFRE, with the fused variant occurring more frequently.^4^ In most cases, the ectopic kidney is positioned inferior to the orthotopic kidney and is commonly associated with renal fusion. CFRE represents a rare congenital malformation resulting from abnormal embryological development of the urinary system. This anomaly has traditionally been classified into six morphological subtypes: (1) inferior ectopia, in which the ectopic kidney is located below the normally positioned kidney; (2) superior ectopia, characterized by fusion of the upper pole of the normally positioned kidney with the lower pole of the ectopic kidney; (3) sigmoid or S-shaped configuration; (4) lump or pancake kidney; (5) L-shaped kidney; and (6) disc-shaped kidney.^5^ However, Zhuo Yin et al. subsequently described an additional morphological variant, termed the “Y-type” crossed-fused renal ectopia, in which fusion of the ureters is observed.^6^

Renal fusion anomalies are of considerable clinical relevance, as approximately half of affected individuals develop associated complications, including urinary obstruction, recurrent UTIs, and nephrolithiasis, as observed in the present case. These sequelae are primarily attributable to abnormal renal positioning and aberrant vascular anatomy, which may compromise effective urinary drainage from the collecting system, thereby increasing susceptibility to urinary tract infection and stone formation.^7^ Furthermore, the presence of complex and variable anatomical configurations can render the diagnosis and management of these complications particularly challenging.

The diagnosis of CFRE can be established preoperatively using a combination of imaging modalities, including whole abdominal USG, plain radiography of the kidneys–ureters–bladder (KUB), retrograde pyelography, and three-dimensional computed tomography (3D-CT). USG is useful as an initial investigation to demonstrate the absence of a kidney from its normal anatomical position. KUB radiography and retrograde pyelography further assist in delineating the size, number, and anatomical location of associated calculi. Particular emphasis should be placed on 3D-CT imaging, as detailed visualization of aberrant renal vasculature is crucial for surgical planning, facilitating safe access to the collecting system while minimizing the risk of hemorrhage and other procedure-related complications.^8^

Owing to the extreme rarity of this congenital anomaly, standardized guidelines for the management of associated urolithiasis or malignancy have not been established. Nevertheless, an increasing body of evidence indicates that minimally invasive techniques are effective in treating this condition when complicated by nephrolithiasis, UPJO, or renal carcinoma.^9^ The management strategy for urolithiasis in CFRE is primarily determined by the renal vascular anatomy and stone burden. In asymptomatic patients, conservative management with careful observation and regular follow-up is advisable, as many individuals may remain symptom-free throughout their lifetime. Therapeutic options for urolithiasis in CFRE include open surgery, extracorporeal shock wave lithotripsy (ESWL), percutaneous nephrolithotomy (PCNL), retrograde intrarenal surgery (RIRS), laparoscopic approaches, and flexible ureteroscopic management via retrograde ureteroscopy. With ongoing advances in surgical techniques and instrumentation, minimally invasive procedures have become increasingly feasible and widely adopted, leading to a progressive decline in the use of open surgery.^10^

The index case was an otherwise healthy child presenting with nonspecific abdominal complaints and multiple visits to general practitioners without a definitive diagnosis or treatment. In resource-limited settings, where routine medical checkups are not commonly practiced, many conditions remain undiagnosed and untreated. This is particularly critical for urogenital anomalies, such as renal ectopia and urinary tract calculi, where post-stone removal follow-up is essential. Inadequate monitoring, coupled with poor nutrition, may contribute to recurrent stone formation and ultimately increase the risk of progression to end-stage renal disease. In settings where advanced minimally invasive techniques are unavailable and pediatric urolithiasis care is limited, open surgical approaches, such as nephrolithotomy, continue to be a safe and effective option for managing stones, including in cases of renal ectopia. Early diagnosis, precise surgical planning, attentive postoperative care, and attention to adequate hydration and balanced nutrition are optimal care of children with complex anomalies, such as crossed fused renal ectopia complicated by renal calculi.

## 4 Conclusion

Sigmoid crossed-fused renal ectopia is a rare congenital anomaly that may occasionally present with pediatric urolithiasis. Detailed preoperative imaging is essential for defining the anatomy and guiding safe management. In selected cases with complex or uncertain anatomy, open nephrolithotomy remains a safe and effective treatment option with favorable outcomes on follow-up.

## CRediT authorship contribution statement

**Turyalai Hakimi:** Writing – review & editing, Writing – original draft, Visualization, Validation, Supervision, Software, Resources, Project administration, Methodology, Investigation, Funding acquisition, Formal analysis, Data curation, Conceptualization. **Edris Nejrabi:** Methodology. **Zamaryalai Hakimi:** Validation, Project administration, Investigation. **Mansoor Aslamzai:** Methodology. **Mohammad Anwar Jawed:** Visualization, Resources.

## Consent to publish

Written informed consent was obtained from the patient's parents for publication of this case report and accompanying images. A copy of the written consent is available for review by the Editor-in-Chief of this journal on request.

## Conflict of interest

The authors have no conflict of interest.

## Guarantor

The corresponding author is the guarantor of the work, having the responsibility of data access and controlling the decision to publish.

## Funding

No fund and grant.

## References

[1] Amin Q.K., Arshad S., Anthony N., Yousafzai Z.A., Arshad S. (2021). Case report on crossed fused renal ectopia with a large calculus and its management. Cureus.

[2] Naseri M. (2016). Cross-fused renal ectopia associated with vesicoureteral reflux: a case report. J Ren Inj Prev.

[3] Resorlu M., Kabar M., Resorlu B. (2015). Retrograde intrarenal surgery in cross-fused ectopic kidney. Urology.

[4] Huang L., Lin Y., Tang Z. (2018). Management of upper urinary tract calculi in crossed fused renal ectopic anomaly. Exp Ther Med.

[5] Liu L., Yang J., Zhu L. (2010). Crossed-fused renal ectopia associated with inverted-Y ureteral duplication, ectopic ureter, and bicornuate uterus. Urology.

[6] Yin Z., Yang J.R., Wei Y.B. (2014). A new subtype of crossed fused ectopia of the kidneys. Urology.

[7] Amin M.U., Khan S., Nafees M. (2009). Crossed fused renal ectopia with staghorn calculus and gross hydronephrosis. J Coll Physician Surg Pak.

[8] Cakmak O., Isoglu C.S., Peker E.A. (2016). Renal cell carcinoma in a patient with crossed fused renal ectopia. Arch Ital Urol Androl.

[9] Giani A., Garancini M., Delitala A. (2017). 3D-laparoscopic anterior rectal resection in a patient with crossed fused renal ectopia: the importance of 3D imaging. Minerva Chir.

[10] Kumar S., Singh S., Jain S. (2015). Robot-assisted heminephrectomy for chromophobe renal cell carcinoma in L-shaped fused crossed ectopia: a surgical challenge. Korean J Urol.

